# Pneumocystis jirovecii Pneumonia in a Patient With Newly Diagnosed HIV and a High CD4 Count

**DOI:** 10.7759/cureus.46680

**Published:** 2023-10-08

**Authors:** Michelle Koifman, Bhavyakumar Vachhani, Krishna S Haridasan, Mossammat Mansur

**Affiliations:** 1 Internal Medicine, The Brooklyn Hospital Center, New York, USA; 2 Infectious Disease, The Brooklyn Hospital Center, New York, USA

**Keywords:** human immuno-deficiency virus (hiv), acquired immune deficiency syndrome (aids), pneumocystis jiroveci pneumonia prophylaxis, cd4 t-cells, opportunist infections in hiv, pneumocystis jiroveci pneumonia

## Abstract

*Pneumocystis jirovecii* pneumonia (PCP) is a rare, life-threatening opportunistic fungal infection that rarely occurs with CD4 counts greater than 200 cells/mm3. We present a case of PCP in a young male who presented with fever, weakness, dyspnea, and a non-productive cough. He was initially treated for community-acquired pneumonia but was then noted to be HIV positive with signs of immunosuppression such as oral thrush. The CD4 count was found to be very high, at 646 cells/mm3 and 281 cells/mm3 on repeat. The patient was treated with trimethoprim/sulfamethoxazole (TMP/SMX) and fluconazole and further started on highly active antiretroviral therapy (HAART) with TMP/SMX as a means of secondary prophylaxis in the outpatient setting.

## Introduction

The immunocompromised status of patients with undiagnosed or untreated HIV leads to many opportunistic infections. *Pneumocystis jirovecii *pneumonia (PCP) is one of these life-threatening opportunistic fungal infections that is airborne but only affects those that are immunocompromised, and it rarely occurs with CD4 counts greater than 200 cells/mm3. It often presents with signs and symptoms such as tachypnea, dyspnea, cough, and fever. Trimethoprim/sulfamethoxazole (TMP/SMX) is considered the gold standard for prophylaxis against PCP when CD4 counts are less than 200 cells/mm3. It is also considered the preferred drug for PCP treatment [1,2]. We present an interesting case of a young male with an elevated CD4 count on two occasions who was diagnosed with PCP.

## Case presentation

A 32-year-old male with a past medical history of tobacco use presented to the hospital with fever, weakness, dyspnea, and a non-productive cough that had been ongoing for one week. He reported being vaccinated with the COVID-19 vaccine and denied any recent sick contacts. Vital signs were significant for a fever of 38.7 degrees Celsius, a heart rate of 105 beats per minute, a respiratory rate of 20 breaths per minute, and an oxygen saturation of 90% on room air. The arterial blood gas was noted to have a pH of 7.45, a partial pressure of carbon dioxide (pCO2) of 40, a partial pressure of oxygen (pO2) of 91, a bicarbonate (HCO3) of 28, and an oxygen (O2) saturation of 97 on 2 L of nasal cannula. The alveolar-arterial gradient (A-a gradient) was 58.6 mm Hg. A physical examination revealed mild respiratory distress. Initial laboratory findings showed no leukocytosis, slight anemia, elevated inflammatory markers, and no lactic acidosis. The laboratory findings are documented in Table 1.

**Table 1:** Pertinent laboratory values on initial presentation

Test	Value	Reference Range
White cell count	7.4 K/cmm	4.8-10.8 K/cmm
Hemoglobin	12.3 g/dL	13.1-15.5 g/dL
Erythrocyte sedimentation rate	80 MM/HR	0-15 MM/HR
C-reactive protein	64.43 mg/L	<5.00 mg/L
Lactate dehydrogenase	538 U/L	125-220 U/L
Lactic acid	1.4 MMOL/L	0.5-2.2 MMOL/L

**Figure 1:**
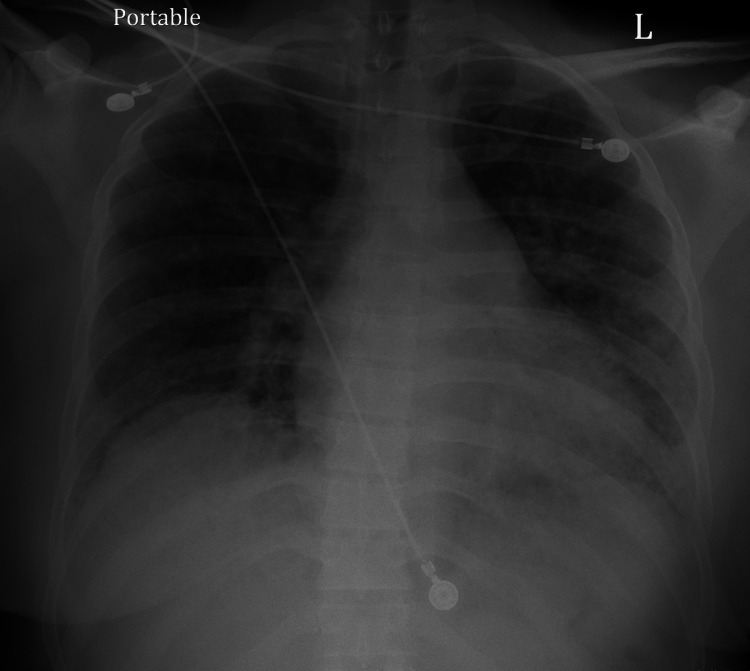
Anterior-posterior chest X-ray showing moderate pulmonary vascular congestion

**Figure 2:**
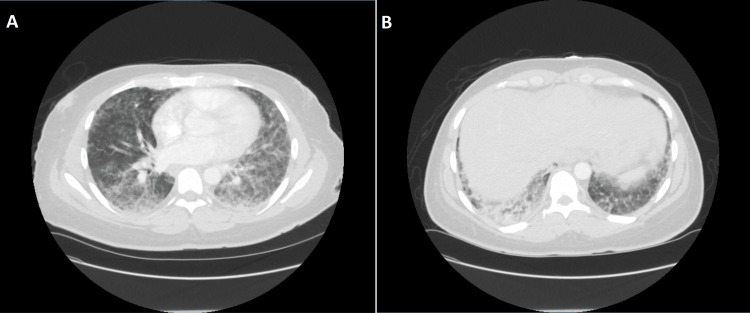
CTA chest A: Extensive diffuse bilateral ground-glass opacities; (B) Confluent region of consolidation in the right posterior lower lobe compatible with an infectious or inflammatory process CTA: Computed tomography angiography

The COVID-19 and influenza tests were negative. A septic workup, including urinalysis, blood cultures, a methicillin-resistant *Staphylococcus aureus* (MRSA) swab, and respiratory culture, was collected. An electrocardiogram (EKG) showed sinus tachycardia. A chest radiograph (chest X-ray) showed pulmonary vascular congestion (Figure 1). The computed tomography angiography (CTA) chest with contrast was negative for pulmonary embolism but showed diffuse bilateral ground-glass opacities with a more confluent region of consolidation in the right posterior lower lobe (Figure 2).

The patient was started on ceftriaxone and azithromycin for community-acquired pneumonia coverage. Further laboratory values were collected (Table 2), including the HIV antigen/antibody test, HIV viral load, and an absolute CD4 lymphocyte count. The infectious disease team noted oral thrush and recommended intravenous TMP/SMX as a treatment for possible PCP due to signs of immunosuppression despite the high CD4 count. The patient was also started on fluconazole and nystatin suspensions. Prednisone was also started due to the elevated A-a gradient. As the respiratory culture grew* S. aureus*, the patient was also started on linezolid as per infectious disease recommendations while pending sensitivities. The remainder of the septic workup was negative.

**Table 2:** Further laboratory values

Test	Value	Reference Range
HIV antigen/antibody combo	Reactive	Nonreactive
HIV viral load	697000 copies/mL	Not detected
Absolute CD4 cells	646 cells/mm3	490-1740 cells/mm3

**Figure 3:**
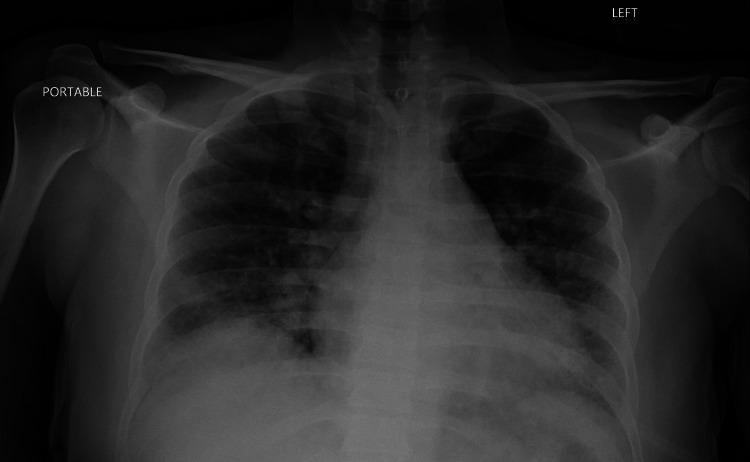
Anterior-posterior chest X-ray showing no significant change in diffuse bilateral patchy opacities compared to the initial chest radiograph on presentation

The repeat CD4 count two days later was 281 cells/mm3, and the repeat chest X-ray showed no significant change in the diffuse opacities, which did not fit with the presentation (Figure 3). The final growth of the respiratory culture did not show MRSA, and linezolid was further discontinued. Pulmonology performed bronchoscopy with bronchoalveolar lavage (BAL), and PCP was confirmed by Gomori methenamine silver (GMS) stain. Gastroenterology did not recommend endoscopic evaluation as symptoms of dysphagia had improved. On discharge, the patient was given three weeks of oral TMP/SMX double strength (DS) and fluconazole for esophageal candidiasis. The patient was also given a prednisone taper. Antiretroviral therapy was not started to avoid immune reconstitution inflammatory syndrome (IRIS). When the patient followed up two weeks later in the outpatient setting, he was started on bictegravir, emtricitabine, and tenofovir alafenamide (Biktarvy), and the dose of TMP/SMX was reduced to the daily prophylactic dose. The patient continued on this dose of TMP/SMX until the HIV viral load was negative.

## Discussion

*Pneumocystis jirovecii* pneumonia is caused by the human pathogen* P. jirovecii*, which is a spherical, oval, cup-shaped, thick-walled cyst that usually measures 6 to 8 μm in diameter and is classified as a fungus [1]. It is transmitted through an airborne route from person to person. Individuals who are immunocompetent may unknowingly carry *P. jirovecii* due to lung colonization and spread it to the immunocompromised. Despite being one of the most common and serious opportunistic infections in patients with AIDS, prophylaxis and highly active antiretroviral therapy (HAART) have reduced its incidence. The TMP/SMX is the gold standard for prophylaxis, and regimens usually include daily and three times a week dosing with single or DS doses that should be initiated when the patient has a CD4 count less than 200 cells/mm3 and a detectable viral load [2,3].

Patients with PCP present with dyspnea, a nonproductive cough, and fatigue, progressing over a few days to weeks. Physical examination may include tachypnea, tachycardia, fever greater than 38.1°C, oral thrush as a co-infection, crackles, and rhonchi. Markers such as lactate dehydrogenase (LDH) are related to prognosis, as non-survivors had a higher LDH. Arterial blood gas in hypoxic patients often shows a high A-a gradient. Chest X-rays can show diffuse bilateral perihilar interstitial infiltrates, and CT chest may show ground glass attenuation [3,4].

Interestingly, one study found that only three (5%) of 61 cases of PCP occurred above a CD4 count of 250 cells/mm3, while another study showed that only four (9%) of 43 patients with PCP had CD4 counts greater than 250 cells/mm3, and 75% of these had CD4 counts that were less than 333 cells/mm3 [5]. In another study with 100 patients, only three (6%) of 49 patients with PCP had CD4 counts greater than 200 cells/mm3 [6]. Our patient had accompanying signs of immunosuppression, which aided in diagnosis and prompted us to consider PCP, despite an initial CD4 count of 646 cells/mm3. In a study with 346 individuals, out of which a total of 168 participants had oral lesions, oral candidiasis was the only lesion found to have a significant association with a CD4 count of less than 350 cells/mm3 and the only lesion significantly predictive of immunosuppression [7]. The repeat CD4 count in the case of our patient was 281 cells/mm3. On repeat six months later in the outpatient setting, the CD4 count was 262 cells/mm3.

Clinicians need to be vigilant for opportunistic infections such as PCP, even with high CD4 counts, to avoid potential morbidity. Nevertheless, it is still important to account for the potential reason there have been other higher-than-expected CD4 counts in the literature in addition to this case. For instance, it has been found that there is a diurnal variation in the CD4 count, with the lowest counts being in the morning and the highest in the evening [8]. It can also be influenced by any factors that lead to an increase or decrease in the WBC count, such as infection, medications, or other chronic conditions. More specifically, leukocytosis may increase the CD4 count, while leukopenia may result in a decreased count. A patient's immunological status may also not be accurately reflected due to factors such as laboratory variability that may falsely increase or decrease CD4 counts [9].

Demonstration of PCP through histopathology or cytopathology is necessary for diagnosis through either sputum induction or bronchoscopy with BAL [10]. The preferred treatment includes TMP/SMX for 21 days. For patients with gas exchange abnormalities receiving antibiotics for less than 72 hours, corticosteroids improve survival. Following PCP treatment, TMP-SMX is recommended as a secondary prophylaxis. If the patient is on HAART, has an undetectable viral load, and has a CD4 count that is greater than 200 cells/mm 3 for at least three months, secondary prophylaxis can be discontinued [11,12]. In our case, TMP/SMX was later discontinued as a secondary prophylaxis due to an undetectable viral load.

## Conclusions

This case of PCP demonstrates the importance of considering many differential diagnoses that could contribute to a patient’s symptoms. It highlights the importance of paying close attention to the entire patient presentation for early detection of life-threatening opportunistic infections in susceptible patients. Aside from what is known to contribute to either the increase or decrease in the CD4 count, such as the time of day, WBC count, medications, chronic conditions, or lab variability, the reason why such anomalies may occur still needs to be further explored, as there are limited case reports on this.

## References

[1] Hughes WT (1996). Pneumocystis Carinii. Medical Microbiology, 4th edition.

[2] Rivera MJG, Ciofoaia GA (2023). Pneumocystis jirovecii Prophylaxis. https://www.ncbi.nlm.nih.gov/books/NBK560530/.

[3] Truong J, Ashurst JV (2022). Pneumocystis jirovecii Pneumonia. https://www.ncbi.nlm.nih.gov/books/NBK482370/.

[4] Boudes P, Furhman C, Verra F, Sobel A (1990). Value of the LDH level in pneumocystis carinii pneumonia in patients infected with human immunodeficiency virus. Ann Med Interne (Paris).

[5] Jung AC, Paauw DS (1998). Diagnosing HIV-related disease: using the CD4 count as a guide. J Gen Intern Med.

[6] Masur H, Ognibene FP, Yarchoan R (1989). CD4 counts as predictors of opportunistic pneumonias in human immunodeficiency virus (HIV) infection. Ann Intern Med.

[7] Nanteza M, Tusiime JB, Kalyango J, Kasangaki A (2014). Association between oral candidiasis and low CD4+ count among HIV positive patients in Hoima Regional Referral Hospital. BMC Oral Health.

[8] Hull MW, Harris M, Montaner JSG (2017). Principles of Management of HIV in the Industrialized World. Infectious Diseases, 4th Edition.

[9] Li R, Duffee D, Gbadamosi-Akindele MF (2023). CD4 Count. https://www.ncbi.nlm.nih.gov/books/NBK470231/.

[10] Shibata S, Kikuchi T (2019). Pneumocystis pneumonia in HIV-1-infected patients. Respir Investig.

[11] Sullivan A, Lanham T, Krol R, Zachariah S (2020). Pneumocystis jirovecii pneumonia in an HIV-infected patient with a CD4 count greater than 400 cells/μL and atovaquone prophylaxis. Case Rep Infect Dis.

[12] Gagnon S, Boota AM, Fischl MA, Baier H, Kirksey OW, La Voie L (1990). Corticosteroids as adjunctive therapy for severe Pneumocystis carinii pneumonia in the acquired immunodeficiency syndrome. A double-blind, placebo-controlled trial. N Engl J Med.

